# Exploring leukocyte differential count ratio profiles as inflammatory biomarkers in diabetic retinopathy: a systematic review and meta-analysis

**DOI:** 10.1186/s12886-025-04075-y

**Published:** 2025-05-01

**Authors:** Ohisa Harley, Yufilia Suci Amelia, Elsa Gustianty, Nanny N. M. Soetedjo, Arief S. Kartasasmita

**Affiliations:** 1 Doctoral Program in Medical Sciences, Faculty of Medicine, Padjadjaran University, Terusan Ekologi- No 7, Ir. Soekarno Street Km 21, Jatinangor, Bandung, West Java 45363 Indonesia; 2 Netra Eye Clinic, Sumatera Street No. 46 - 68, Bandung, West Java 40114 Indonesia; 3 Departement of Ophthalmology, Faculty of Medicine, Padjadjaran University, Ir. Soekarno Street Km 21, Bandung, West Java 45363 Indonesia; 4 Departement of Endocrinology and Internal Medicine, Faculty of Medicine, Padjadjaran University, Ir. Soekarno Street Km 21, Jatinangor, Bandung, West Java 45363 Indonesia; 5 Cicendo Hospital National Eye Center, Cicendo Street No.4, Babakan Ciamis, Bandung, West Java 40117 Indonesia

**Keywords:** Diabetic retinopathy, Neutrophil, Lymphocyte, Monocyte, Platelet, Biomarker

## Abstract

**Background:**

Diabetic retinopathy (DR) is increasingly prevalent and a major cause of irreversible blindness, particularly in developing countries. Limited access to ophthalmologists often leads to delayed diagnosis, emphasizing the need for more affordable and widely accessible screening methods to facilitate early identification. Recently, several studies have demonstrated variability in findings regarding the relationship between leukocyte differential count ratio biomarkers and DR. This study aims to investigate the association between leukocyte differential count ratios—NLR (Neutrophil-to-Lymphocyte Ratio), PLR (Platelet-to-Lymphocyte Ratio), MLR (Monocyte-to-Lymphocyte Ratio), and SII (Systemic Immune-Inflammation Index)—and the stages of diabetic retinopathy (DR).

**Methods:**

A comprehensive literature search was conducted across several databases up to September 2024, with a focus on identifying studies examining the relationship between the leukocyte differential count ratio profiles and diabetic retinopathy. Review Manager was used to conduct the meta-analyses. The Newcastle Ottawa Scale (NOS) were used to assess the included studies.

**Results:**

A total of 38 studies were included in the systematic review and 27 studies were included in the meta-analysis. The mean differences in the NLR and PLR values were significantly different among the groups and were higher in the PDR group (0.68 (95%CI 0.42–0.95, *p* < *0.05*) and 19.57 (95%CI 10.68–28.46, *p* < *0.05*; respectively). These findings were followed by significant differences in SII value 202.53 (95% CI 196.19–208.86*, p* < *0.05).* Moreover, the MLR values were not significantly different among the groups (*p* > *0.05*).

**Conclusion:**

NLR, PLR, and SII are associated with both the presence and progression of DR, with increasing levels of NLR and PLR reflecting a higher risk and severity of the disease. However, it is still necessary to justify the need to combine them with other clinical parameters to confirm the diagnosis.

**Supplementary Information:**

The online version contains supplementary material available at 10.1186/s12886-025-04075-y.

## Introduction

Diabetic retinopathy (DR) is a leading cause of irreversible blindness, with its prevalence rising alongside the increasing burden of diabetes [1, 2]. In many developing countries, limited access to retina specialists leads to late-stage diagnoses, heightening the risk of vision loss. Early detection is crucial for preventing complications, yet access to ophthalmologic screening remains a challenge, particularly in resource-limited settings. Therefore, there is an urgent need for accessible and cost-effective biomarkers to facilitate early DR identification and risk stratification [2].

Inflammation plays a critical role in DR pathogenesis, contributing to endothelial dysfunction, microvascular damage, and increased vascular permeability [3, 4]. Previous studies have reported elevated levels of inflammatory cytokines such as IL- 6 and TNF-α in DR patients [5]. However, these biomarkers require specialized laboratory facilities, limiting their practicality for routine screening [6, 7].

Routine blood parameters, including leukocyte differential count ratios, have emerged as potential inflammatory biomarkers due to their availability, cost-effectiveness, and widespread use in clinical practice [8–11]. Integrates neutrophils, platelets, and lymphocytes, provides a broader reflection of systemic inflammation and immune status. Among them, the neutrophil-to-lymphocyte ratio (NLR), platelet-to-lymphocyte ratio (PLR), monocyte-to-lymphocyte ratio (MLR), and systemic immune-inflammation index (SII) have been shown as better predictive value than differential count alone for inflammatory and vascular diseases, including diabetes. [8–11].

In recent years, several studies have reported elevated levels of NLR, PLR, MLR and SII in DR patients [10, 12–14]. However, the findings remain inconsistent. The clinical relevance of these biomarkers in DR diagnosis and progression is still debated. A comprehensive evaluation through meta-analysis is essential to determine whether these biomarkers can be effectively utilized in DR. This study aims to systematically review and analyze the association between NLR, PLR, MLR, and SII with DR through meta-analysis, evaluating their potential as screening and management tools, particularly in regions with limited ophthalmologic access.

## Methods

### Protocol and registration

This systematic review was carried out according to the Preferred Reporting Items for Systematic Review and Meta-analysis (PRISMA) Guidelines and registered in PROSPERO (CRD42024596414).

### Eligibility criteria and outcomes of interest

Research studies could be considered for inclusion if they met the following criteria.

1. Population: Type 2 Diabetes Melitus (T2DM) patients with a history of diabetic retinopathy with or without diabetic macular edema
2. Intervention/Exposure: Leukocyte Differential Count Ratio (NLR, PLR, MLR, and SII)
3. Control: Healthy controls or diabetic patients without diabetic retinopathy. If a study includes both, only diabetic patients without retinopathy are included in the analysis.
4. Outcome: The data are presented as the mean value with standard deviation (SD) for the NLR, PLR, MLR and SII
5. Designs: Randomized controlled trial (RCTs), prospective and retrospective studies, case–control studies, case series, and cross-sectional studies. We also consider other designs if the data are represented.

For exclusion criteria were as follows: (1) Other types of diabetes and (2) combination or unspecified microvascular complications in the study groups.

The NLR was defined as the ratio of neutrophils to lymphocytes. The PLR was defined as the ratio of platelets to lymphocytes. MLR was defined as the ratio of monocytes to lymphocytes. SII was defined as the neutrophil × platelet/lymphocyte count.

### Search strategy

We employed medical subject headings (MeSH) and free text terms related to diabetic retinopathy and systemic peripheral blood markers to identify related studies. We searched various databases, including PubMed, EBSCO, and ProQuest. The search strategy included the following terms: ["Diabetic Retinopathy"[MeSH Term] OR"Diabetic Complications"[MeSH Term] OR"Diabetic Retinopathy"[Text Word] OR"Diabetic Complications"[Text Word] OR"Diabetic Microvascular Complications"] AND ["Complete Blood Count"[MeSH Term] OR"Blood Cells"[MeSH Term] OR"Neutrophils"[MeSH Term] OR"Monocytes"[MeSH Term] OR"Platelets"[MeSH Term] OR"Lymphocytes"[MeSH Term] OR"Peripheral Blood Marker"[Text Word] OR"Neutrophil to Lymphocyte Ratio"[Text Word] OR"Monocyte to Lymphocyte Ratio"[Text Word] OR"Platelet to Lymphocyte Ratio"[Text Word] OR"Systemic Immune Inflammation Index"[Text Word]]. We also manually examined the reference lists of the included research and relevant reviews, searched Google Scholar to identify any potentially relevant articles. The exploration involved synonyms and variations of the terms ‘diabetic retinopathy’ and ‘peripheral blood marker’. (see Supplementary File 1). We limited our search to articles published in English and full text. Incomplete data or missing data were excluded.

### Data selection, collection and extraction

We managed the identified studies using the Mendeley reference manager. Initially, the studies will undergo a process of deduplication, and then they will be screened based on their titles and abstracts to assess their eligibility criteria. Two authors (OH and YSA) independently carried out this screening. In the event of any disagreements during the selection process or quality assessment, these issues were discussed with other authors to reach a consensus (EG, NS, ASK). Relevant data were extracted to perform a qualitative synthesis. The extracted data included the author, year of study, study design, number of participants, eligibility criteria, stages of diabetic retinopathy (if any), systemic peripheral blood marker, and statistical value.

### Risk of bias assessment

The Newcastle Ottawa Scale (NOS) was used to evaluate the quality of the case–control, cross-sectional, and cohort studies. A study's overall score of 7–9 indicated a low risk of bias, a score of 5–6 indicated some concerns or a moderate risk of bias, and a score of < 5 indicated a high risk of bias (Fig. 7).

### Data analysis

A comprehensive qualitative analysis were conducted to provide a summary and explanation of the characteristics of the included studies. Moreover, this synthesis explores the relationships among studies. We performed a meta-analysis using the random effects model. The overall impact assessment involves the analysis of continuous data using mean differences. To assess statistical heterogeneity, we utilized the I^2^ statistic *(p* < *0.05* or I^2^ ≥ 50). The relevant information was merged and calculated using the statistical software Review Manager version 5.4.

### Sensitivity analysis and publication bias

We conducted sensitivity analyses using multiple approaches to evaluate the reliability of the meta-analysis results. Subgroup analyses were performed for NPDR and PDR groups. Additionally, leave-one-out analyses were carried out to assess the impact of individual studies on the overall pooled estimate by systematically excluding one study at a time. Meta-regression was also performed to examine potential sources of variability. Publication bias was assessed through visual inspection of funnel plot asymmetry, complemented by Egger’s test and the Trim-and-Fill method for further statistical validation.

## Results

### Baseline characteristics

**Fig. 1:**
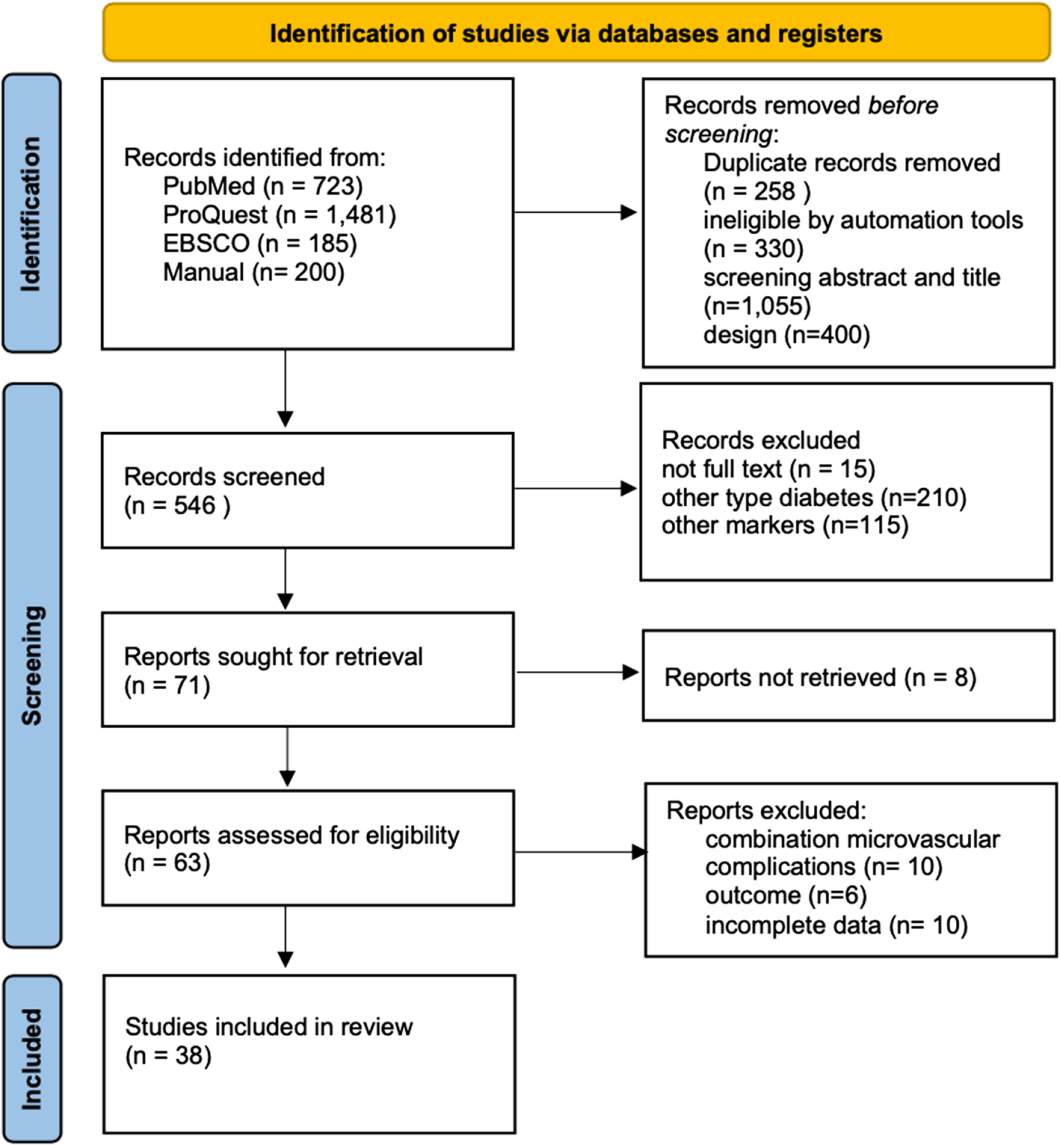
PRISMA diagram flow

A total of 2.589 studies were identified through database searches and manual exploration (Fig. 1). After removal, 546 studies underwent initial screening based on their titles and abstracts. Out of these, 63 studies were further assessed to determine their eligibility criteria. A total of 38 studies were included for final review (Fig. 1).

A total of 27 eligible studies were in the pooled analysis. The total number of participants in our studies included 11,930 individuals with T2DM. The included studies revealed that hematologic biomarkers such as the NLR, MLR, PLR, and SII related to the presence of diabetic retinopathy complications in patients with T2DM. For a comprehensive overview of the study characteristics, refer to Table 1.

**Table 1:** Characteristics of included study

No.	Author, Year	Design Study	Age (mean ± SD, years)	Population	Diagnosis of DR	DR groups	Differential Count Ratio Profile	Findings
1	Abdullah, 2021 [15]	case–control	DR 7.36 ± 8.35; no DR 55.24 ± 10.83	India	ICDR severity scales	no DR and DR	NLR	NLR were higher in DR group
2	Akdogan, 2016 [16]	retrospective cohort	59.8 ± 9.2	Turkey	dilated funduscopy	control, DM without DR, DM with DR	NLR and PLR	PLR were higher in DR compared to control. No difference in NLR group
3	Atli, 2022 [17]	cross-sectional	NPDR 61.44 ± 6.25; PDR 60.86 ± 4.70; no DR 56.15 ± 10.03; control 43.10 ± 7.82	Turkey	digital fundus based on ETDRS	control, no DR, NPDR and PDR	NLR and PLR	higher NLR and PLR in PDR group
4	Bhattacharyya, 2021 [18]	cross-sectional	59.89 ± 10.9	India	ICDR severity scale	DM with nephropathy, neuropathy, and retinopathy	NLR	NLR higher in DM with more than one microvascular complication
5	Chittawar, 2017 [19]	cross-sectional	51.12 ± 11.28	India	comprehensive assessment include dilated funduscopy and fundus FA based on ETDRS	DM only	NLR	Higher quartile NLR (2.60–6.28) increase DR incidence
6	Cardoso, 2021 [20]	prospective cohort	60.0 ± 9.6	Brazil	comprehensive assessment includes dilated funduscopy, OCT macula based on ICDR severity scale	microvascular DR and macrovascular complications	NLR, PLR, MLR	no hematological parameter was predictive of any microvascular outcome including retinopathy
7	Ciray, 2015 [21]	cross-sectional	59.7 ± 11.3	Turkey	dilated funduscopy based on ICDR severity scale	NPDR and PDR	NLR	Higher NLR was not associated with DR and severity of DR
8	Dascalu AM, 2023 (a) [11]	retrospective cohort	no DR 66.9 ± 5.76; NPDR 66.4 ± 6.07; PDR 62.0 ± 10.59	Romania	Visual Evoked Potentials	no DR, NPDR, PDR	NLR, PLR	Higher NLR associated with PDR. PLR were not different statistically among groups
9	Dascalu AM, 2023 (b) [10]	retrospective cohort	65.6 ± 8.9	Romania	examination microdots, blot hemorrhage, hard exudates, soft exudates, and new vessel formation	no DR, NPDR, PDR	NLR, PLR, MLR, SII	Higher NLR, MLR, MPV, and SII associated with PDR group. PLR were not associated with DR
10	Dogan, 2024 [22]	retrospective cohort	no DR 53.1 ± 6.9; NPDR 54.3 ± 7.1; PDR 55.7 ± 5.9; control 53.8 ± 7.4	Turkey	comprehensive examination include dilated fundus and fundus photography	control, no DR, NPDR and PDR	NLR, MLR, PLR, SII	NLR, PLR and SII were higher in NPDR and PDR groups. MLR were not significantly higher in NPDR or PDR group
11	El-Tawab, 2023 [23]	case–control	DR 50.48 ± 7.98; control 52.47 ± 8.85	Egypt	N/A	PDR and no PDR	NLR	NLR associated with DR
12	Fawwad, 2018 [24]	retrospective cohort	no DR 52.33 ± 11.05; microvascular complications 55.86 ± 10.72	Pakistan	digital retina photographs	no DR and DR	NLR	NLR associated with DR
13	Gao Y, 2024 [25]	retrospective cohort	no DR 53.78 ± 1.88; NPDR 51.72 ± 1.64; PDR 53.00 ± 1.52	China	fundus photography, FFA, and OCT based on ICDR severity scale	no DR, NPDR, PDR	NLR, PLR, SII	NLR, PLR and SII were associated with DR and DR stages
14	He X, 2022 [26]	cross-sectional	61.3 ± 13.2	US	comprehensive asessment based on ETDRS and ICDR severity scale	no DR and DR	NLR	NLR associated with DR
15	Huang Q, 2021 [27]	cross-sectional	61 ± 13.85	China	N/A	control, DM without DR, NPDR, PDR,	MLR	MLR were higher in PDR group
16	Ilhan C, 2019 [28]	Prospective case–control	Control 62.68 ± 10.40; NPDR 61.14 ± 9.33; PDR 59.63 ± 7.07	Turkey	based on IAO in 2002	control, severe NPDR, PDR	NLR, MLR, PLR	NLR associated with DR. MLR and PLR were not different among groups
17	Ilhan C, 2020 [29]	prospective cohort	control 63.54 ± 5.68; DME 58.22 ± 11.35; non-DME 61.92 ± 6.82	Turkey	fundus examination	control, NPDR with DME, NPDR without DME	NLR, MLR, PLR	NLR and MPV/L were higher in DME group. MLR and PLR were not different among groups
18	Lei C, 2023 [30]	cross-sectional	55.46 ± 10.08	China	medical records	PDR with DME, PDR without DME	NLR, PLR, MLR, SII	NLR, PLR, LMR, and SII were not associated with CMT/DME
19	Li J, 2024 [13]	cross-sectional	54.67 ± 12.86	China	non-mydriatic fundus photography based on ICDR severity scale	no DR and DR	NLR, PLR, SII	NLR, PLR and SII were associated with DR
20	Mahajan, 2023 [31]	prospective cohort	56.3 ± 13.24	India	dilated funduscopy based on ICDR severity scale	DR	NLR	Higher NLR in DR group. NLR associated with increased risk of microvascular complications
21	Moursy, 2015 [32]	retrospective cohort	DR 56.19 ± 7.27; no DR 53.60 ± 6.15; control 53.50 ± 6.66	Egypt	based on Global DR Project Group	no DR, NPDR, PDR	NLR	NLR were significantly higher in NPDR and PDR group compared to no DR. NLR were not significantly higher in PDR compared to NPDR
22	Ozturk, 2013 [33]	cross-sectional	DR 66.60 ± 4.20; no DR 66.78 ± 4.12	Turkey	non-mydriatic fundus photography based on ETDRS	no DR and DR	NLR	NLR were higher in diabetic group with complications
23	Rajendrakumar AL, 2023 [34]	retrospective cohort	61.7 ± 12.7	Scottish	based on ICDR severity scale	developed DR, death without DR, no DR	NLR	NLR associated with DR
24	Sari, 2021 [35]	case–control	N/A	Indonesia	based on ICDR severity scale	no DR, NPDR, PDR	NLR, PLR, MLR	NLR and PLR were not significantly higher in DR. MLR were insignificantly lower in DR compared to control
25	Tang Y, 2024 [36]	retrospective cohort	no DR 47.3 ± 11.2; DR 52.4 ± 10.9	China	dilated fundus, FA, and SD-OCT	no DR and DR	NLR	NLR associated with DR
26	Ulu SM, 2013 [37]	cross-sectional	control 48.38 ± 5.45; DM 50.31 ± 5.20	Turkey	undilated fundus	control, DM without DR, DM with DR	NLR	Higher NLR associated with DR and correlated with DR grades
27	Wan H, 2020 [38]	cross-sectional	67 ± 9	China	non-mydriatic fundus photography by ophthalmologist based on ICDR severity scale	DR, NPDR, PDR	NLR	Higher NLR were not associated with prevalence of DR
28	Wang H, 2022 [14]	cross-sectional	63.8 ± 10.8	US	dilated funduscopy and OCT based on ETDRS	no DR, NPDR, PDR	MLR	MLR were associated with PDR
29	Wang JR, 2020 [39]	cross-sectional	no DR 55.44 ± 11.27; DR 56.48 ± 9.86	China	dilated color fundus photography	no DR and DR	NLR, PLR, MLR	NLR and PLR associated with DR. MLR were not associated with DR
30	Wang RT, 2015 [40]	cross-sectional	control 58.7 ± 5.9; no DR 60.3 ± 6.0; DR 66.6 ± 5.8	China	N/A	control, DM without DR, DM with DR	NLR	NLR were higher in DR group
31	Wang S, 2023 [41]	cross-sectional	no DR 59 ± 4.25 DR 60, 3.43	China	N/A	no DR and DR	SII	SII were higher in DR groups and associate dwith DR
32	Xiaodong L, 2023 [42]	cross-sectional	57.8 ± 10.52	China	N/A	NPDR, PDR, NPDR with DN, and PDR with DN	NLR and PLR	NLR and PLR were higher in DR with DN compared to DR group only
33	Yanxia C, 2024 [43]	retrospective cohort	early 59.06 ± 11.74; advanced 60.34 ± 9.65; severe 61.37 ± 8.59; atrophic 61.71 ± 13.11	China	N/A	early, advanced, severe and atrophic DME	NLR, MLR, PLR, SII	SII and the decline in SRF and HRF ≥ were associated with DME stages
34	Yeter DY, 2022 [44]	retrospective cohort	63 ± 8.5	Turkey	N/A	DR without DME, DR with DME, DM without DR/DME	NLR and MHR	NLR and MHR were associated with DME
35	Yue Song, 2015 [45]	case–control	no DR 55.75 ± 11.11; NPDR 53.31 ± 10.56; PDR 56.00 ± 8.89	China	funduscopy based on ISO	DM without DR, NPDR, PDR	NLR, MLR, PLR	Higher NLR and PLR in DR group. Only MLR were associated with DR independently
36	Zeng J, 2022 [46]	retrospective cohort	no DR 55.23 ± 10.19; NPDR 57.69 ± 9.56; PDR 55.83 ± 8.18	China	Medical records	control, NPDR, PDR	NLR, MLR, PLR	NLR, PLR, MLR were higher in DR groups. Only PLR were associated with DR risk independently
37	Zhang P, 2021 [47]	cross-sectional	DR 68.33 ± 8.40; no DR 68.10 ± 8.47	China	dilated fundus	no DR and DR	NLR	NLR were higher in DR group
38	Zhu Y, 2022 [48]	retrospective cohort	no-DME 56.38 ± 10.51; DME 56.00 ± 8.25	China	OCT based on ESASO classification	severe DR with DME, severe DR without DME	NLR, MLR, PLR	NLR, PLR, MLR, MHR was not different among groups

*DME* Diabetic Macular Edema, *DR* Diabetic Retinopathy, *FFA* Fundus Fluorescein Angiography, *ICDR* International Clinical Diabetic Retinopathy and Diabetic Macular Edema Disease, *IAO* International Academy of Ophthalmology, *ISO* International Society of Ophthalmology, *MLR* Monocyte-to-lymphocyte Ratio, *NLR* Neutrophil-to-lymphocyte Ratio, *OCT* Optical Coherence Tomography, *PLR* Platelet-to-lymphocyte Ratio, *NPDR* Non-Proliferative Diabetic Retinopathy, *PDR* Proliferative Diabetic Retinopathy; SII: Systemic Immune-Inflammation Index

*N/A* Not available

### NLR and DR

**Fig. 2:**
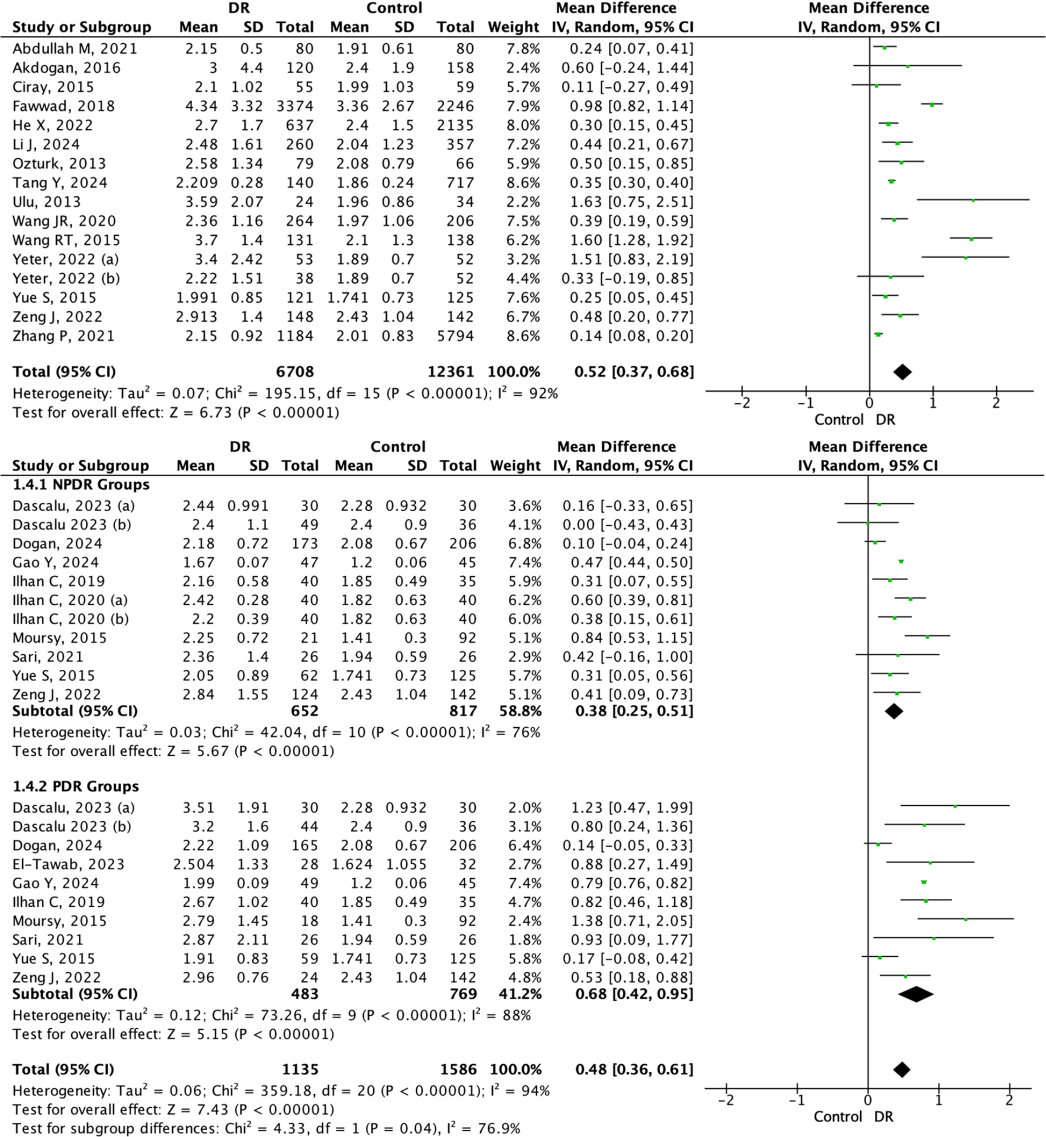
Forrest plot and subgroup analysis of NLR in NPDR and PDR groups. NLR: Neutrophil-to-lymphocyte Ratio; NPDR: Non-Proliferative Diabetic Retinopathy; PDR: Proliferative Diabetic Retinopathy

Sixteen studies were included in the meta-analyses of the NLR and DR (Fig. 2). The analyses revealed a mean difference of 0.52 (95% CI: 0.37–0.68, *p* < 0.05) with high heterogeneity (I^2^ = 92%, *p* < 0.05). These results suggest a significant difference and association between a higher NLR and DR. Furthermore, we analyzed the value of the NLR in the NPDR and PDR groups, involving nine and ten studies, respectively. The overall mean difference between NPDR and PDR was statistically significant at 0.48 (95%CI: 0.36–0.61, *p* < 0.05). In the subgroup analysis, the NPDR group had a mean difference of 0.38 (95%CI: 0.25–0.51, *p* < 0.05), and the PDR group showed a mean difference of 0.68 (95%CI 0.42–0.95, *p* < 0.05) compared to control. These results consistently indicate a higher value of NLR associated with the stages of DR, which also indicates that the PDR stages is greater than the NPDR stage.

### PLR and DR

**Fig. 3:**
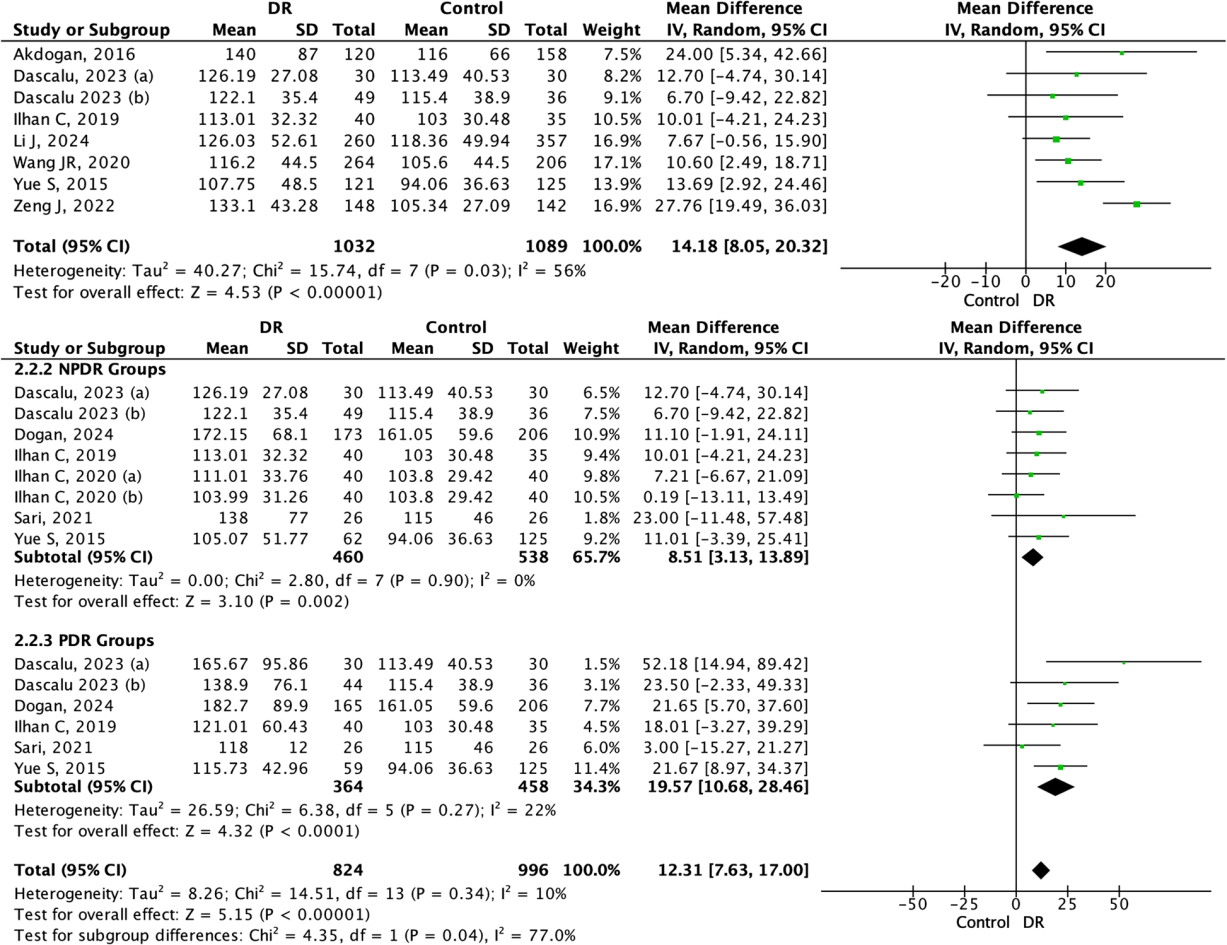
Forrest plot and subgroup analysis of PLR in NPDR and PDR groups. PLR: Platelet-to-lymphocyte Ratio; NPDR: Non-Proliferative Diabetic Retinopathy; PDR: Proliferative Diabetic Retinopathy

For the PLR and DR meta-analyses, we included a total of eight studies in the DR group, seven studies in the NPDR group, and six studies in the PDR group (Fig. 3). There was a mean difference of 12.31 (95% CI: 7.63–17.00, *p* < 0.05) with low heterogeneity (I^2^ = 10%, *p* < 0.05) in the DR group. Furthermore, for the DR stages, the overall mean difference effect was consistent with the mean difference results. According to the subgroup analyses, the NPDR group presented a mean difference of 8.51 (95%CI: 3.13–13.89*, p* < 0.05), followed by the PDR group, which presented a mean difference of 19.57 (95%CI: 10.68–28.46, *p* < 0.05) (Fig. 6A and B). These results suggest that a higher PLR is associated with stages of DR.

### MLR and DR

**Fig. 4:**
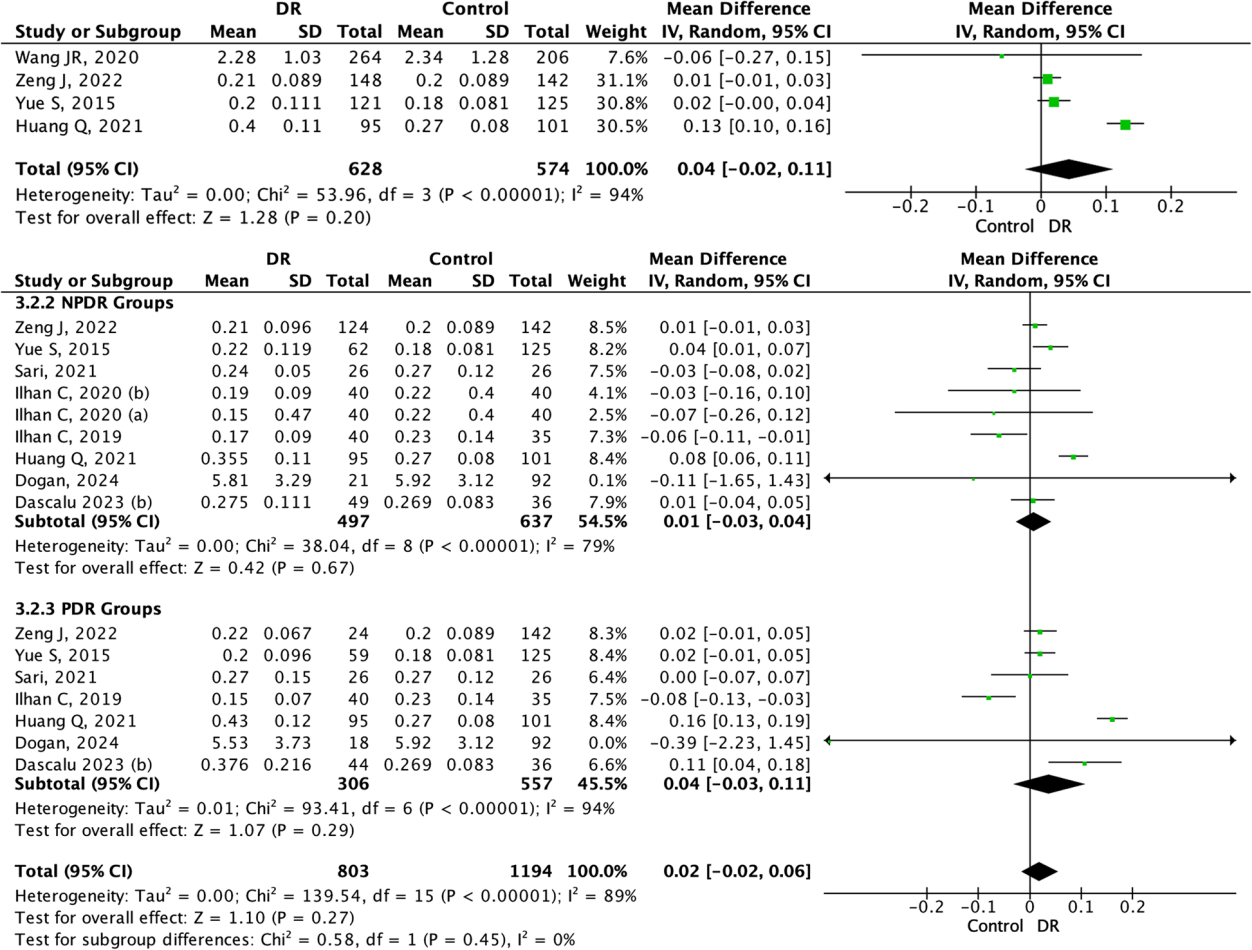
Forrest plot and subgroup analysis of MLR in NPDR and PDR groups. MLR: Monocyte-to-lymphocyte Ratio; NPDR: Non-Proliferative Diabetic Retinopathy; PDR: Proliferative Diabetic Retinopathy

In the MLR and DR meta-analyses, we included four studies for DR, eight studies for NPDR and seven studies for the PDR group (Figs. 4 ). The DR group analysis revealed an insignificant mean difference, showing consistent results in subgroup analysis (0.02 (95% CI − 0.02–0.06, *p* > *0.05*)).

**Fig. 5:**
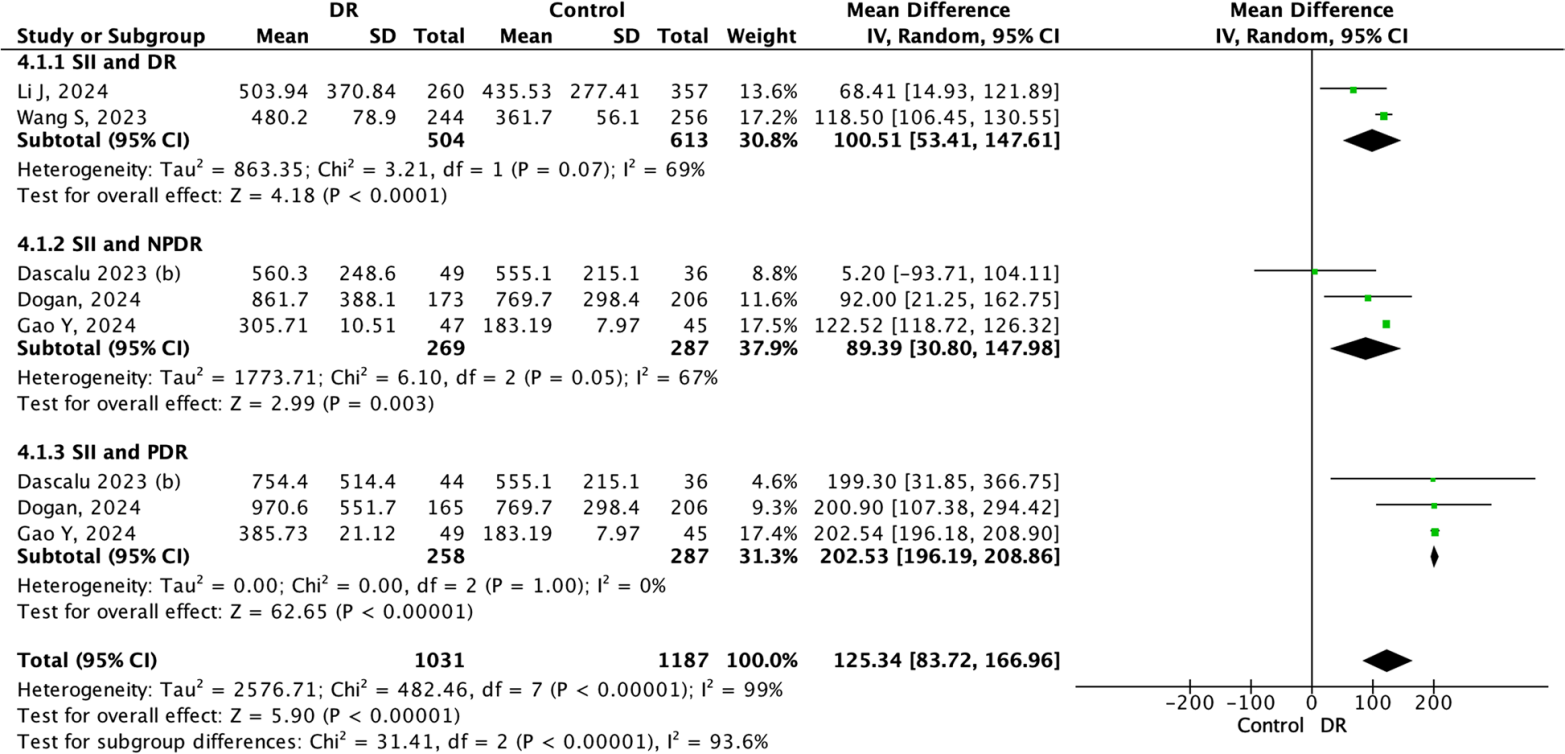
Forrest plot and subgroup analysis of SII in NPDR and PDR groups. Systemic immune-inflammation index; NPDR: Non-Proliferative Diabetic Retinopathy; PDR: Proliferative Diabetic Retinopathy

### SII and DR

Five studies included in DR and subgroup analyses. The forest plot of the SII revealed that the value of the mean difference in the SII differed across DR stages. In the NPDR group, the value of mean difference was 89.39 (95%CI 30.80–147.98*, p* < *0.05*) greater than that in the control. In the PDR group, the mean difference was 202.53 (95% CI 196.19–208.86*, p* < *0.05*) was higher compared to control. The overall mean difference effects were significant in two groups with significant tests for subgroup differences (I^2^ = 99%, *p* < *0.05*). However, small study plots may had small clinical effects (Fig. 5).

### Risk of bias assessment

**Fig. 6:**
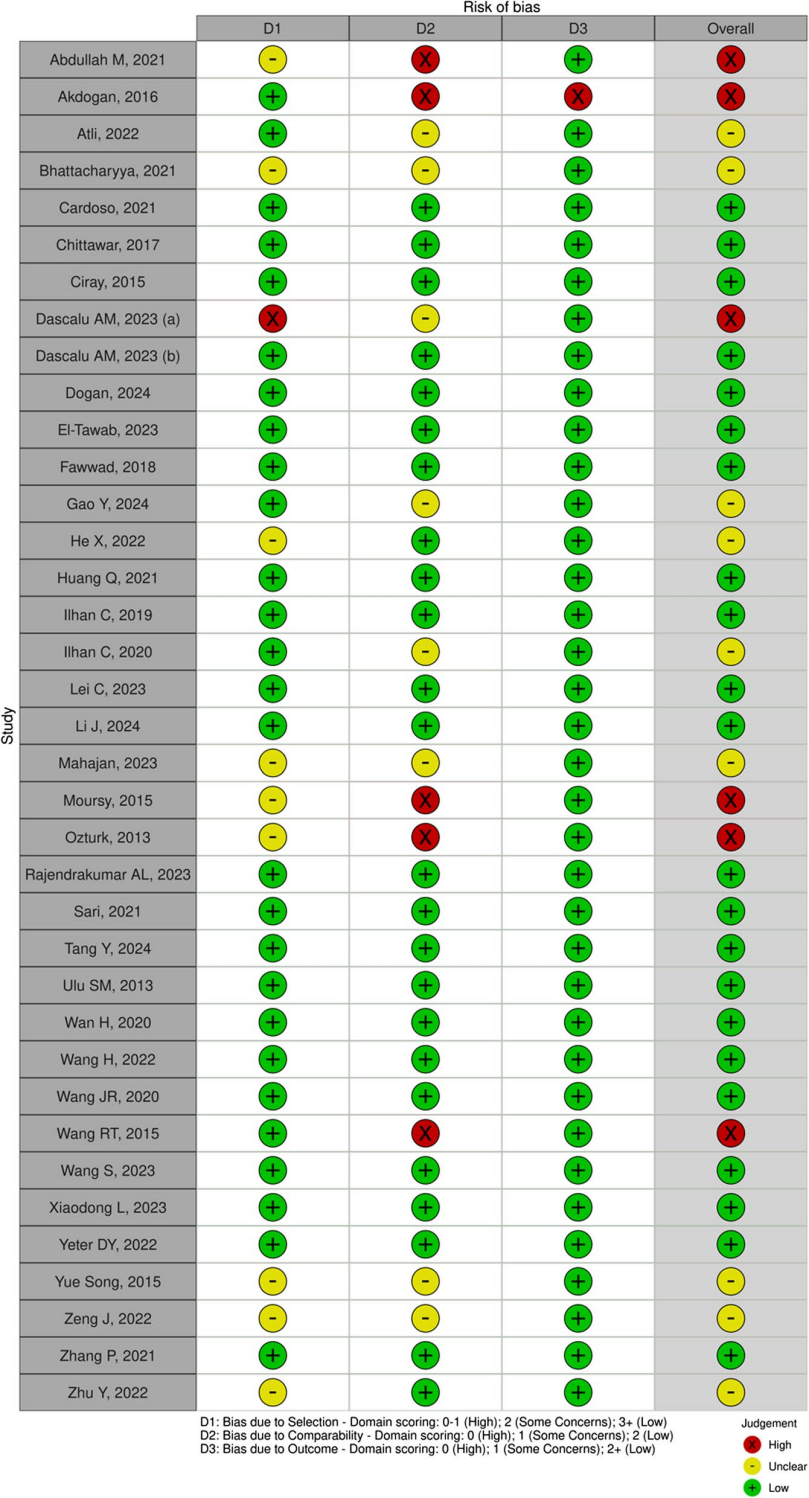
Risk of bias of included study

**Fig. 7:**
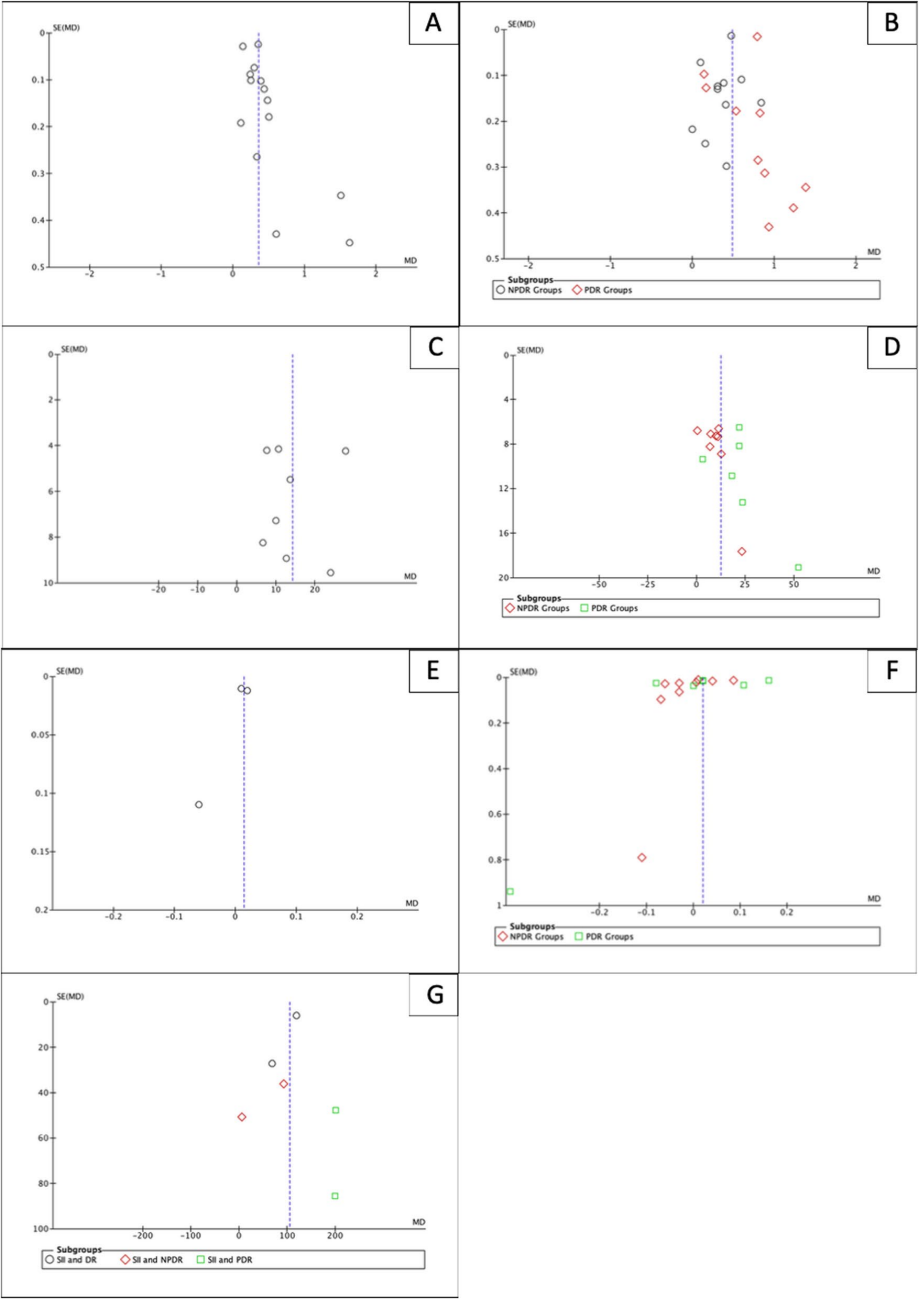
Funnel plots. **A** Funnel plots of NLR in DR. **B** Funnel Plots of NLR in NPDR and PDR. **C** Funnel plots of PLR in DR. **D** Funnel Plots of PLR in NPDR and PDR. **E** Funnel plots of MLR in DR. **F** Funnel Plots of MLR in NPDR and PDR. **G** Funnel Plots of SII in DR, NPDR and PDR. MLR: Monocyte-to-lymphocyte Ratio; NLR: Neutrophil-to-lymphocyte Ratio; PLR: Platelet-to-lymphocyte Ratio; NPDR: Non-Proliferative Diabetic Retinopathy; PDR: Proliferative Diabetic Retinopathy

The quality assessment of the 38 included studies identified six studies with a high risk of bias and nine studies with a moderate risk of bias (Fig. 6). Funnel plots suggest a possibility of publication bias (Fig. 7). Figure 7A–D show slight asymmetry in the funnel plots, supporting the possibility of publication bias in pooled NLR and PLR studies. Figure 7E and F show asymmetry in the funnel plots and a small-study effect in MLR studies. Figure 7G indicates symmetry in the funnel plots, but this may be insignificant due to the small number of SII studies.

### Sensitivity analysis and publication bias

To ensure the reliability of our findings, we conducted a Trim-and-fill analysis and Egger’s regression test to assess and adjust for potential publication bias in our meta-analysis. For NLR, Egger’s regression test indicated significant publication bias (t = 2.90, df = 19, *p* = 0.009). However, the intercept test was not significant (R2 = 30.62%, t = 1.54, *p* = 0.139), suggesting that small-study effects may not be the primary cause of funnel plot asymmetry. The initial random-effects model reported an MD of 0.585 (95% CI: 0.566–0.605, *p* < 0.05), which was adjusted to an MD of 0.485 (95% CI: 0.357–0.613, *p* < 0.05) using the Trim-and-Fill method, with no studies trimmed. Despite the persistence of significant heterogeneity (*p* < 0.05), the adjusted model provides a more balanced estimate of the NLR value.

For PLR, Egger’s regression test also indicated publication bias (t = 4.21, df = 13, *p* = 0.001), while the intercept test was not significant (R2 = 59.58%, t = 1.32, *p* = 0.21). The initial model estimated a pooled proportion of 12.192 (95% CI: 7.809–16.575, *p* < 0.05), which was adjusted to 11.324 (95% CI: 6.187–16.496, *p* < 0.05) after adding two hypothetical studies via the Trim-and-Fill method. The adjusted model also provides a more balanced PLR value; however, the heterogeneity remained insignificant (*p* < 0.05).

For MLR studies, Egger’s regression test revealed publication bias (t = − 5.05, df = 13, *p* = 0). However, both the initial random-effects model and the Trim-and-Fill method yielded insignificant pooled effect results (*p* = 0.157 and *p* = 0.859, respectively), with high heterogeneity (*p* = 0.001).

For SII studies, the initial random-effects model estimated an MD of 141.489 (95% CI: 138.352–144.626, *p* = 0), which was adjusted to an MD of 125.341 (95% CI: 83.723–166.959, *p* = 0) using the Trim-and-Fill method. This result indicates an unbalanced pooled estimate of the SII value with high heterogeneity. Due to the limited number of studies, Egger’s regression intercept test could not be conducted.

**Fig. 8:**
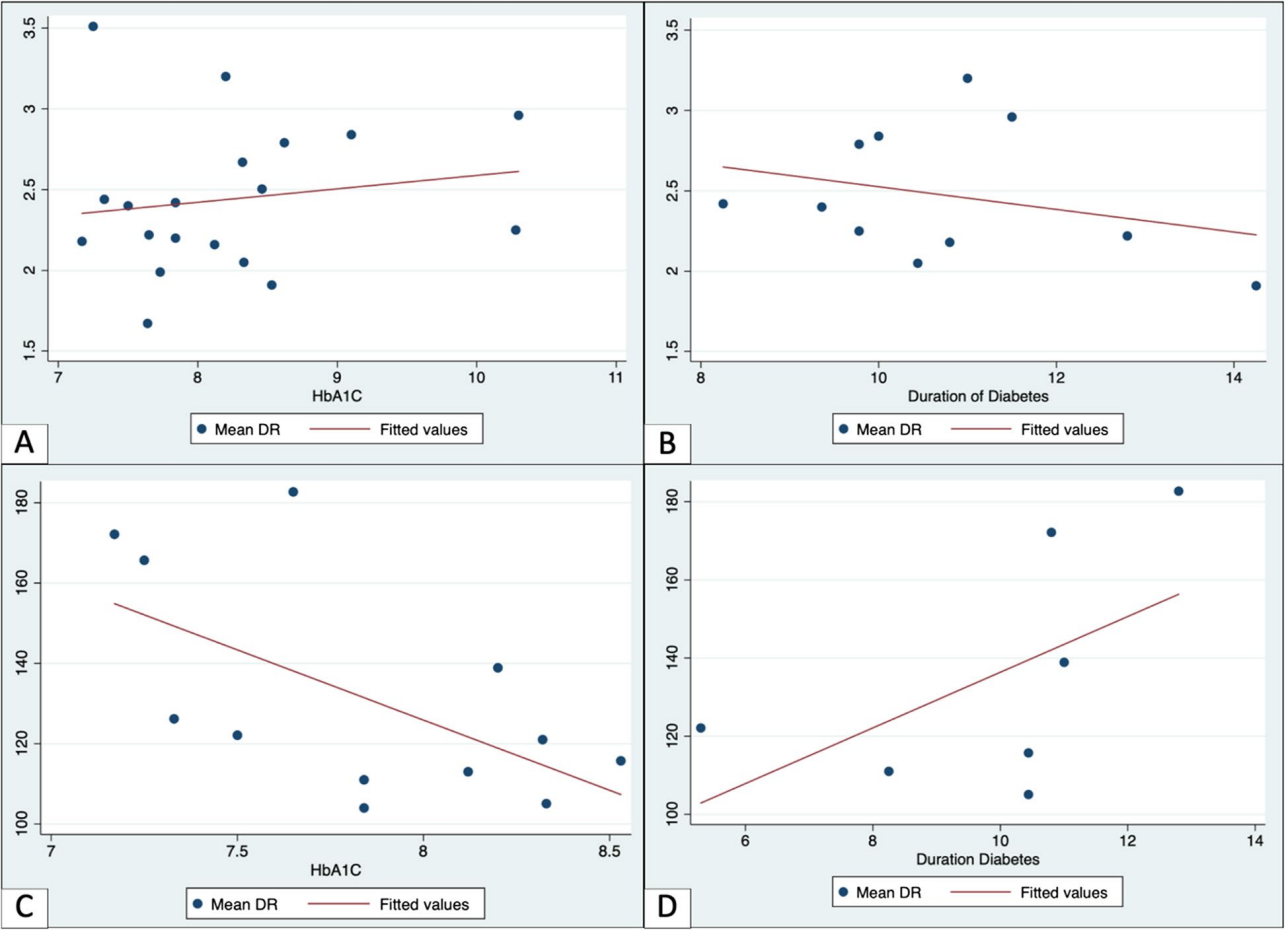
Meta-regression analysis in DR subgroup. **A** and **B** Meta-regression of HbA1c and Duration of Diabetes based on NLR values. The regression line indicates a slight positive trend between HbA1c and NLR, while showing a slight negative trend between diabetes duration and NLR. **C** and **D** Meta-regression of HbA1c and Duration of Diabetes based on PLR values. The regression line demonstrates a negative trend between HbA1c and PLR, whereas it exhibits a positive trend between diabetes duration and PLR. HbA1c: Hemoglobin A1 C; NLR: Neutrophil-to-lymphocyte Ratio; PLR: Platelet-to-lymphocyte Ratio

Additionally, we performed a meta-regression analysis to examine the relationship between mean NLR and PLR in DR subgroups and two independent variables: HbA1c levels and diabetes duration. HbA1c showed a weak positive but statistically insignificant association with NLR (β = 0.2643, 95% CI: − 0.1589 to 0.6876, *p* = 0.205), with an adjusted R-squared value indicating that approximately 30% of the variability in mean DR is explained by mean HbA1c levels. Similarly, diabetes duration exhibited a positive but insignificant association with NLR (β = 2.9475, 95% CI: − 17.3061 to 23.2010, *p* = 0.227), with an adjusted R-squared value indicating 59.58% variability (Fig. 8 and Table 2).

**Table 2:** Effect measures of leukocyte ratio profiles and additional markers in the pooled meta-analysis

No.	Author, Year	Duration of diabetes (years)	HbA1 C	Creatine (mg/dl)	CRP (mg/L)	OR (95%CI) of NLR	OR (95%CI) of PLR	OR (95%CI) of MLR	OR (95%CI) of SII
1	Abdullah, 2021 [15]	11.6 ± 3.25	no DR 6.8 ± 0.4; DR 8.13 ± 0.7	N/A	N/A	N/A	N/A	N/A	N/A
2	Akdogan, 2016 [16]	15.8 ± 7.3	no DR 9.2 ± 2.6; DR 9.3 ± 2.0	N/A	N/A	N/A	N/A	N/A	N/A
3	Ciray, 2015 [21]	9.1 ± 6.5	no DR 8.7 ± 2.4; DR 9.7 ± 2.1	DR: 42.2 ± 797 No DR: 54.7 ± 115	DR 6.64 ± 6.01 no DR 4.78 ± 5.46	N/A	N/A	N/A	N/A
4	Dascalu AM, 2023 (a) [11]	> 5 years	no DR 7.01 ± 0.972; NPDR 7.33 ± 1.59; PDR 7.25 ± 0.978	no DR: 0.865 ± 0.215; NPDR 0.97 ± 0.323; PDR 1.3 ± 1.03	N/A	2.01 (1.29–3.14)*	1.01 (1–1.02)*	N/A	N/A
5	Dascalu AM, 2023 (b) [10]	No DR 5.3 ± 2.4; NPDR 9.36 ± 3.3; PDR 11 ± 3.1	No DR 7.2 ± 1.1; NPDR 7.5 ± 1.8; PDR 8.2 ± 1.8	no DR 0.9 ± 0.3; NPDR 1 ± 0.5; PDR 1.3 ± 0.8	N/A	1.645 (1.189–2.275)*		1.662 (1.209–2.284)*	1.001 (1–1.003)*
6	Dogan, 2024 [22]	no DR 8.61 ± 4.22; NPDR 10.8 ± 3.13; PDR 12.8 ± 4.38	no DR 6.09 ± 2.99; NPDR 7.17 ± 2.63; PDR 7.65 ± 3.79	N/A	no DR 0.54 ± 0.38; NPDR 0.64 ± 0.29; PDR 0.86 ± 0.31	N/A	N/A	N/A	N/A
7	El-Tawab, 2023 [23]	N/A	no PDR 7.27 ± 1.28; PDR 8.46 ± 1.66;	N/A	N/A	3.312 (1.262–8.696)*	N/A	N/A	N/A
8	Fawwad, 2018 [24]	no DR 10.89 ± 7.38; DR 15.47 ± 8.47	no DR 9.14 ± 2.27; DR 9.71 ± 2.42	no DR 1.10 ± 0.65; DR 1.35 ± 0.99	N/A	1.766 (1.789–2.093)*	N/A	N/A	N/A
9	Gao Y, 2024 [25]	N/A	no DR 6.90 ± 0.19; NPDR 7.64 ± 1.18; PDR 7.73 ± 0.24	N/A	N/A	1.122 (0.200–2.043)*	0.038 (0.018–0.058)*	N/A	0.007 (0.001–0.01)*
10	He X, 2022 [26]	no DR 10.1 ± 4.9; DR 12.9 ± 3.0	no DR 7.4 ± 1.8; DR 7.9 ± 1.9	N/A	no DR 0.5 ± 0.7; DR 0.6 ± 0.8	1.076 (1.015, 1.142)*	N/A	N/A	N/A
11	Huang Q, 2021 [27]	N/A	N/A	N/A	control 2.26 ± 1.77 no DR 6.18 ± 4.39 DR 10.31 ± 6.64	N/A	N/A	5.302 (2.925–15.201)	N/A
12	Ilhan C, 2019 [28]	N/A	control 5.26 ± 0.44; NPDR 8.12 ± 1.09; PDR 8.32 ± 1.07	N/A	N/A	N/A	N/A	N/A	N/A
13	Ilhan C, 2020 [29]	PDR DME 8.25 ± 4.83; PDR non DME 6.58 ± 2.72	7.84 ± 0.87	N/A	N/A	N/A	N/A	N/A	N/A
14	Li J, 2024 [13]	9.32 ± 7.10	No DR 8.77 ± 2.67 DR 8.00 ± 2.00	No DR 0.646 ± 0.160 DR 0.982 ± 0.976	N/A	1.93 (1.10–3.40)*	1.47 (1.14–2.03)*	N/A	1.47 (1.14–2.03)*
15	Moursy, 2015 [32]	no DR 10.21 ± 5.74; DR 9.78 ± 7.40	DR 10.28 ± 2.50; no DR 8.62 ± 2.63	no DR 1.41 ± 0.30; DR 2.52 ± 1.21	N/A	N/A	N/A	N/A	N/A
16	Ozturk, 2013 [33]	no DR 6.15 ± 3.22 DR 8.44 ± 4.62;	no DR 9.28 ± 2.54; DR 9.93 ± 2.30	no DR 0.80 ± 0.62; DR 0.97 ± 0.88	no DR 25.72 ± 25.40; DR 15.26 ± 14.14	1.904 (1.170–3.100)*	N/A	N/A	N/A
17	Sari, 2021 [35]	N/A	N/A	N/A	N/A	2.765 (1,045–7,315)*	1 (1–1)	0,00 (0,00–1,49)	N/A
18	Tang Y, 2024 [36]	no DR 46.75 ± 32.41 DR 87.25 ± 43.17	no DR 7.50 ± 1.93; DR 8.10 ± 2.19	no DR 0.72 ± 0.18 DR 0.70 ± 0.20	N/A	1.292 (1.112–1.501)*	N/A	N/A	N/A
19	Ulu SM, 2013 [37]	7.33 ± 7.16	N/A	0.78 ± 0.31	no DR 0.82 ± 0.55; DR 2.59 ± 2.58	N/A	N/A	N/A	N/A
20	Wan H, 2020 [38]	10.75 ± 3.25	N/A†	N/A†	N/A†	DR 1.09 (0.82–1.45) NPDR (1.06 (0.80–1.42) PDR 0.94 (0.23–3.86)	N/A	N/A	N/A
21	Wang JR, 2020 [39]	no DR 66.16 ± 66.25; DR 122.86 ± 87.38 months	no DR 9.68 ± 2.66; DR 9.96 ± 2.45	no DR 0.78 ± 0.20; DR 0.95 ± 0.53	N/A	1.37 (1.06–1.78)*	1.05 (0.99- 1.11)	0.96 (0.82–1.13)	N/A
22	Wang RT, 2015 [40]	no DR 2.7 ± 1.7; DR 8.6 ± 1.7	no DR 6.9 ± 1.1; DR 7.7 ± 0.7	N/A	N/A	N/A	N/A	N/A	N/A
23	Wang S, 2023 [41]	DR 14,2.75 no DR 7, 2.93	no DR 8.46 ± 2.43; DR 8.81 ± 1.76	no DR: 0.72 ± 0.14 DR 0.72 ± 0.14	N/A	N/A	N/A	N/A	1.002 (1.000–1.004)*
24	Yeter DY, 2022 [44]		no DR 8.09 ± 1.9; DR 8.3 ± 1.7	N/A	N/A	4.004 (1.656–9.685)*	N/A	N/A	N/A
25	Yue Song, 2015 [45]	no DR 5.38 ± 3.02; NPDR 10.44 ± 2.94 PDR 14.25 ± 3.94	no DR 7.33 ± 2.22; NPDR 8.33 ± 2.22; PDR 8.53 ± 1.93	No DR 60.00 ± 14.07 NPDR 58.75 ± 18.79 PDR 60.50 ± 24.44	N/A	NS	NS	54.574 (2.708–1099.907)*	N/A
26	Zeng J, 2022 [46]	No DR 2.00 ± 1.52; NPDR 10.00 ± 2.25 PDR 11.50 ± 3.00	No DR 9.10 ± 0.88 NPDR 9.10 ± 0.95 PDR 10.30 ± 0.72	No DR 56.40 ± 4.99 NPDR 60.50 ± 6.14 PDR 60.45 ± 8.46	N/A	N/A	1.020 (1.010–1.029)*	NS	N/A
27	Zhang P, 2021 [47]	DR 14.41 ± 5.82; no DR 11.65 ± 5.50	no DR 7.10 ± 1.42; DR 8.00 ± 1.76	no DR 1.55 ± 0.94 DR 1.52 ± 0.90	N/A	1.132 (1.053–1.217)*	N/A	N/A	N/A

*CRP* C-reactive protein, *DME* Diabetic Macular Edema, *DR* Diabetic Retinopathy, *MLR* Monocyte-to-lymphocyte Ratio, *NLR* Neutrophil-to-lymphocyte Ratio, *OR (95%CI)* Odd Ratio (95% Confidence Interval), *PLR* Platelet-to-lymphocyte Ratio, *NPDR* Non-Proliferative Diabetic Retinopathy, *PDR* Proliferative Diabetic Retinopathy, *SII* Systemic Immune-Inflammation Index

*N/A* not available

^*^Statistically significant (*p* < *0.05*)

^**†**^ Data presented in quartile groups

## Discussion

The use of differential count ratio profiles as inflammatory markers in DR has been widely discussed. Biomarkers such as the NLR, PLR, MLR, and SII are easily accessible and cost-effective. However, their specificity and clinical applicability remain subjects of ongoing investigation. While some studies highlight their potential utility in reflecting systemic inflammation and disease progression, others emphasize the influence of confounding factors, including comorbidities and individual variations in immune response [5, 12, 49, 50]. This meta-analysis aims to elucidate their relevance in DR by synthesizing data from multiple studies.

The key findings in our meta-analysis demonstrate that NLR, PLR, and SII were significantly elevated in patients with DR, with the highest values observed in those with PDR. These results suggest a potential association between systemic inflammation and DR severity. In contrast, MLR did not exhibit a consistent difference among study groups, indicating its limited utility as a biomarker for DR progression.

The precise mechanisms linking differential count ratio profiles to DR pathogenesis remain incompletely understood. DR is recognized as a multifactorial disease, with chronic inflammation playing a critical role in its development. Several systemic inflammatory markers, including CRP and interleukin, have been associated with DR, reflecting the persistent inflammatory state that contributes to retinal microvascular damage. Hyperglycemia-induced oxidative stress and endothelial dysfunction may underlie the observed alterations in NLR, PLR, and SII among DR patients.

Previously, HbA1 C have been identified as predictor in DR stages in some studies [51–53]. Our meta-regression analysis showed an insignificant positive association between HbA1c and NLR in DR subgroups, suggesting that while an increase in HbA1c is associated with a rise in NLR values, it may not be the sole determinant of DR stages and does not fully explain the variability across studies. HbA1c may reflects systemic hyperglycemia and indicate ongoing systemic inflammation. However, since HbA1c represents blood glucose levels over the past two months [53], it does not fully represent the chronic nature of DR pathology. Some studies also suggested that even after prolonged normalization of blood glucose levels, inflammatory damage may be irreversible [54, 55]. Additionally, our analysis found an insignificant positive association between the duration of diabetes and PLR in DR subgroups, indicating that a longer duration of diabetes may be linked to increased PLR values, as reported in previous studies. Since diabetes duration is often self-reported, it may introduce bias. Many individuals seek medical attention only after symptoms appear, making it difficult to accurately determine the disease's exact onset.

The findings of this meta-analysis should be interpreted considering the strengths and limitations of the included studies. This study incorporates a comprehensive literature search, rigorous eligibility criteria, and statistical analyses to address potential bias. The use of Egger’s test and trim-and-fill methods adjust for publication bias and enhances reliability of the findings. Moreover, this study provides a comprehensive perspective on their potential role in DR screening and risk stratification. Among these markers, NLR and PLR may be useful for assessing systemic inflammation in DR. The observed elevation in PLR highlights the possible involvement of platelets in inflammation and endothelial dysfunction, although further studies are required to determine its specificity [56, 57]. SII, which integrates neutrophil, platelet, and lymphocyte counts, offers a more comprehensive inflammatory profile and may enhance its utility in DR assessment. In contrast, the limited significance of MLR suggests that monocyte activity alone may not be a primary driver of DR pathogenesis.

Despite the rigorous methodology employed in this meta-analysis, certain limitations must be acknowledged. First, the relatively small number of studies included in specific analysis such as Egger’s test and Meta-regression for SII and MLR. This may reduce the statistical power and restrict the ability to draw the definitive conclusions. Second, although there were slight publication bias, the heterogeneity remained high, particularly in NLR studies. The secondary data in microvascular complication and population may affect the variability in included studies. Furthermore, the development of DME may contribute to variations in study findings since not all studies assess DME status in DR. This could serve as a basis for further exploration of the impact of DME on elevated leukocyte differential count ratio profiles.

Our findings suggest that NLR, PLR, and SII could serve as an accessible and cost-effective adjunct for identifying at-risk patients, particularly in resource-limited settings, although it may not replace existing diagnostic tools. Further research, such as prospective cohort studies are needed to validate their clinical application and to explore their integration with other diagnostic modalities to enhance DR detection and monitoring. This may be an interesting focus for future studies.

## Conclusion

This study showed that NLR, PLR, and SII are associated with both the presence and progression of DR, with increasing levels of NLR and PLR reflecting a higher risk and severity of the disease. We propose that the leukocyte differential count ratio test, particularly the evaluation of the NLR and PLR, may serve as additional practical and cost-effective screening tools for detecting DR in T2DM patients. This particularly valuable in areas with limited access to ophthalmologists. Thus, healthcare can identify high-risk individuals who may benefit from more comprehensive eye examinations. However, it is still necessary to justify the need to combine them with other clinical parameters to confirm the diagnosis.

## Supplementary Information

Supplementary Material 1

## Data Availability

No datasets were generated or analysed during the current study.

## Abbreviations

CRP: C-reactive protein
DR: Diabetic Retinopathy
HbA1c: Glycated Hemoglobin
IL- 6: Interleukin-6
MLR: Monocyte-to-lymphocyte Ratio
MPV: Mean Platelet Volume
NLR: Neutrophil-to-lymphocyte Ratio
NOS: Newcastle–Ottawa Scale
NPDR: Non-Proliferative Diabetic Retinopathy
PDR: Proliferative Diabetic Retinopathy
PDW: Platelet Distribution Width
PLR: Platelet-to-lymphocyte Ratio
PRISMA: Preferred Reporting Items for Systematic Review and Meta-Analysis
SII: Systemic Immune-Inflammation Index
T2DM: Type 2 Diabetes Mellitus
TNF-α: Tumor Necrosis Factor-alpha
