# Supplementary material for: Exploring leukocyte differential count ratio profiles as inflammatory biomarkers in diabetic retinopathy: a systematic review and meta-analysis
Source: BMC Ophthalmol. 2025 May 1;25:265. doi: 10.1186/s12886-025-04075-y (PMC12044949; doi:10.1186/s12886-025-04075-y)
Supplement: Supplementary file 1 — Supplementary Material 1 [file 12886_2025_4075_MOESM1_ESM.pdf]

Supplementary File 1. Search Strategy

Key Concepts	Concept 1<br>Diabetic retinopathy	Concept 2<br>Peripheral blood marker
Controlled vocabulary terms / Subject terms	“Diabetic Retinopathy” [MeSH Term] OR “Diabetic Complications” [MeSH Term]	“Complete Blood Count” [MeSH Term] OR “Blood Cells” [MeSH Term] OR “Neutrophils” [MeSH Term] OR “Monocytes” [MeSH Term] OR “Platelets” [MeSH Term] OR “Lymphocytes” [MeSH Term]
Free text terms / natural language terms	“Diabetic Retinopathy” [Text Word] OR “Diabetic Complications” [Text Word] OR “Diabetic Microvascular complications”	“Peripheral Blood Marker” [Text Word] OR “Neutrophil to Lymphocyte Ratio” [Text Word] OR “Monocyte to Lymphocyte Ratio” [Text Word] OR “Platelet to Lymphocyte Ratio” [Text Word] OR “Systemic Immune Inflammation Index” [Text Word]

Draft Entry PubMED Search

No	Entry	Filter	Total Findings
1.	"Diabetic Retinopathy" [MeSH Term] OR "Diabetic Complications" [MeSH Term] OR "Diabetic Retinopathy" [Text Word] OR "Diabetic Complications" [Text Word] OR "Diabetic Microvascular complications"	None	53,157
2.	"Complete Blood Count" [MeSH Term] OR "Blood Cells" [MeSH Term] OR "Neutrophils" [MeSH Term] OR "Monocytes" [MeSH Term] OR "Platelets" [MeSH Term] OR "Lymphocytes" [MeSH Term] OR "Peripheral Blood Marker" [Text Word] OR "Neutrophil to Lymphocyte Ratio" [Text Word] OR "Monocyte to Lymphocyte Ratio" [Text Word] OR "Platelet to Lymphocyte Ratio" [Text Word] OR "Systemic Immune Inflammation Index" [Text Word]	None	1,017,380
3.	S1 AND S2	None	904
4.	S1 AND S2	Full Text	723

Draft Entry EBSCO Search

No	Entry	Filter	Total Findings
1.	SU ("Diabetic Retinopathy") OR SU ("Diabetic Complications" OR TX "Diabetic Retinopathy" OR TX "Diabetic Complications" OR TX "Diabetic Microvascular complications"	None	129,522

2.	SU ("Complete Blood Count") OR SU ("Blood Cells") OR SU ("Neutrophils") OR SU ("Monocytes") OR SU ("Platelets") OR SU ("Lymphocytes") OR TX "Peripheral Blood Marker" OR TX "Neutrophil to Lymphocyte Ratio" OR TX "Monocyte to Lymphocyte Ratio" OR TX "Platelet to Lymphocyte Ratio" OR TX "Systemic Immune Inflammation Index"	None	750,553
3.	S1 AND S2	None	1,888
4.	S1 AND S2	Full Text	1,481

Draft Entry ProQuest Search

No	Entry	Filter	Total Findings
1.	MAINSUBJECT.EXACT (Diabetic Retinopathy) OR MAINSUBJECT.EXACT (Diabetic Complications) OR fulltext (Diabetic Retinopathy) OR fulltext (Diabetic Complications) OR fulltext (Diabetic Microvascular complications)	None	47,696
2.	MAINSUBJECT.EXACT (Complete Blood Count) OR MAINSUBJECT.EXACT (Blood Cells) OR MAINSUBJECT.EXACT (Neutrophils) OR MAINSUBJECT.EXACT (Monocytes) OR MAINSUBJECT.EXACT (Platelets) OR MAINSUBJECT.EXACT (Lymphocytes) OR fulltext (Peripheral Blood Marker) OR fulltext (Neutrophil to Lymphocyte Ratio) OR fulltext (Monocyte to Lymphocyte Ratio) OR fulltext (Platelet to Lymphocyte Ratio) OR fulltext (Systemic Immune Inflammation Index)	None	1,690
3.	S1 AND S2	None	185
